# Toward precision psychological rehabilitation: predicting CBT efficacy in post-stroke depression using machine learning

**DOI:** 10.3389/fpsyt.2025.1722447

**Published:** 2026-01-06

**Authors:** Jingyuan Lin, Jiansong Yu

**Affiliations:** 1 Department of Rehabilitation Medicine, Fujian Provincial Geriatric Hospital, Fuzhou, China; 2 Department of Rehabilitation Medicine, Taizhou Hospital of Zhejiang Province Affiliated to Wenzhou Medical University, Taizhou, China

**Keywords:** cognitive behavioral therapy (CBT), machine learning, post-stroke depression (PSD), predictive modeling, retrospective analysis

## Abstract

**Objective:**

This study retrospectively examined the potential benefits of cognitive behavioral therapy (CBT) for post-stroke depression (PSD) and developed an interpretable machine learning model to predict individual treatment response.

**Methods:**

Clinical and psychological data from 120 PSD patients receiving CBT and 123 patients in a control group were analyzed. Changes in PHQ-9, GAD-7, and General Self-Efficacy Scale (GSE) scores were compared between groups. Within the CBT cohort, a random forest classifier was trained to predict treatment response and compared with logistic regression and gradient boosting models. SHAP values and ablation analyses were used to assess feature contributions and model interpretability.

**Results:**

Baseline characteristics were comparable between groups. The CBT group showed greater improvement in depressive symptoms than the control group. Among predictive models, the random forest classifier demonstrated the highest performance (AUC = 0.897; accuracy = 0.861). SHAP and ablation analyses consistently highlighted baseline depressive severity (PHQ-9), anxiety (GAD-7), self-efficacy (GSE), and social support (SSRS) as the most influential predictors of CBT response.

**Conclusion:**

CBT was associated with greater improvement in depressive symptoms among patients with post-stroke depression; however, causal inferences should be made cautiously given the retrospective design. The proposed machine learning model shows preliminary promise for predicting treatment response, but further validation in prospective and multi-center studies is needed before clinical implementation.

## Introduction

Post-stroke depression (PSD) is a common neuropsychiatric complication, affecting approximately 39%–52% of stroke survivors and contributing to impaired recovery, reduced quality of life, and increased mortality (1). Although both pharmacological treatments and cognitive behavioral therapy (CBT) can be beneficial, accurately predicting treatment response remains a major clinical challenge. Conventional approaches often fail to account for individual variability, and predictive tools have not been widely implemented in clinical practice (2).

Machine learning (ML) has emerged as a promising approach for PSD prediction and outcome evaluation. However, current ML studies are limited by overfitting, insufficient external validation, and limited interpretability, which hinder their clinical applicability (3) (4). Developing models that are both robust and interpretable is therefore essential to improving precision in clinical decision-making (5).

While several ML-based models have been proposed to predict PSD, many still lack generalizability and external validation (6, 7), and their limited interpretability reduces clinicians’ confidence in automated predictions (6). Moreover, most existing research focuses on identifying the risk of PSD rather than predicting treatment response. Evidence suggests that personalized treatment strategies are crucial for optimizing outcomes, yet factors influencing CBT response in PSD remain insufficiently understood (8–10).

To address these gaps, this study retrospectively evaluates CBT outcomes in PSD patients and develops an interpretable ML model to predict individual treatment response (11). By integrating clinical, psychological, and demographic data, we aim to identify key baseline predictors and improve the transparency and generalizability of the predictive model through interpretability techniques such as SHAP.

Advancing PSD treatment through interpretable ML approaches may contribute to precision medicine in neurology and psychiatry, providing a foundation for more individualized rehabilitation strategies and enabling timely, tailored interventions for stroke survivors.

## Materials and methods

### Study design and ethical considerations

This was a retrospective study of patients diagnosed with post-stroke depression (PSD) at Fujian Provincial Geriatric Hospital. The study protocol was approved by the hospital’s Ethics Committee (Approval No. 20250803). Data were collected from electronic medical records, with all procedures following the principles of the Declaration of Helsinki. Patient confidentiality was strictly maintained throughout the study.

### Participants: inclusion and exclusion criteria

Eligible participants were adults (≥18 years) with a clinical diagnosis of stroke and subsequent depression according to the Diagnostic and Statistical Manual of Mental Disorders (DSM-5) (12). Additional inclusion criteria required complete demographic, clinical, and psychological assessment data. Exclusion criteria were:

Severe cognitive impairment (Mini-Mental State Examination, MMSE < 24);

History of major psychiatric disorders prior to stroke;

Incomplete or missing medical records.

Only patients with confirmed depression diagnosed within six months after stroke onset were included in the analysis. A total of 243 patients were enrolled, with 120 in the CBT group and 123 in the control group.

### Data collection

Demographic variables included age, sex, marital status, educational level, employment status, monthly income, and living arrangements. Clinical characteristics included stroke type, stroke side, stroke severity (NIHSS score), comorbidities (e.g., hypertension, diabetes), and history of psychological or pharmacological treatment.

Psychological and functional assessments included:

Depression: Patient Health Questionnaire-9 (PHQ-9) (13);

Anxiety: Generalized Anxiety Disorder-7 (GAD-7);

Cognitive function: Mini-Mental State Examination (MMSE);

Self-efficacy: Generalized Self-Efficacy Scale (GSE);

Social support: Social Support Rating Scale (SSRS);

Sleep quality: Pittsburgh Sleep Quality Index (PSQI);

Activities of daily living: Barthel Index.

All assessments were performed at baseline (pre-intervention) and repeated after the intervention.

### Diagnosis of post-stroke depression

PSD was diagnosed according to DSM-5 criteria and further confirmed using the PHQ-9 screening tool (13). A PHQ-9 score ≥10 was used as the diagnostic threshold, consistent with previous validation studies demonstrating high sensitivity and specificity for depression screening in post-stroke populations (14). Diagnoses were independently confirmed by experienced neurologists and psychiatrists.

### Intervention: cognitive behavioral therapy protocol

The CBT group received an 8-week standardized intervention delivered by licensed psychologists trained in CBT for depression. Each session lasted approximately 60 minutes and was conducted once weekly, either individually or in groups. Core components of the CBT program included:

Cognitive restructuring of maladaptive thoughts;

Behavioral activation and problem-solving training;

Relaxation techniques and stress management;

Coping strategy development.

The control group received standard care, consisting of pharmacological treatment and routine stroke rehabilitation, but without structured psychological intervention.

### Outcome measures

The primary outcomes were changes in PHQ-9, GAD-7, and GSE scores between baseline and post-intervention. Secondary outcomes included SSRS, PSQI, Barthel Index, and MMSE scores.

### Definition of CBT treatment response

To ensure conceptual clarity, CBT treatment response was defined prior to statistical and machine learning analyses. A responder was operationally defined as a patient achieving a ≥50% reduction in PHQ-9 score from baseline to post-intervention. This threshold reflects a clinically meaningful improvement commonly used in depression treatment research. Patients who did not meet this criterion were classified as non-responders. This binary outcome was used in both traditional regression analyses and the development of the machine learning predictive models.

### Statistical analysis

All statistical analyses were performed using R software (version 4.0.3). Continuous variables were examined for distributional characteristics. Variables following approximately normal distributions were analyzed using independent-sample t-tests, whereas skewed variables—such as time since stroke—were compared using the non-parametric Mann–Whitney U test.

Categorical variables were summarized as counts and percentages, and compared using chi-square (χ²) tests.

To identify independent predictors of CBT response, multivariate logistic regression was performed using the glm() function, adjusting for potential confounders. Model results were expressed as odds ratios (OR) with 95% confidence intervals (CI).

### Data preprocessing

All variables were examined for missing data. No missing values were identified in any demographic, clinical, or psychological variables in the CBT group (0% missingness). Therefore, a complete-case analysis was used for all subsequent modeling. Class imbalance was also assessed, and the distribution of responders versus non-responders was found to be moderate. As a result, no oversampling or undersampling procedures were applied. To maintain class proportions, all train–test splits were conducted using stratified sampling. Model performance was evaluated using AUC and accuracy on the held-out test set. To prevent information leakage, all post-intervention assessment data were strictly excluded from feature engineering. This included post-treatment PHQ-9, GAD-7, GSE, SSRS, PSQI, and MMSE scores, as well as all change-derived variables (e.g., ΔPHQ-9). Only baseline demographic, clinical, and psychological variables collected prior to the initiation of CBT were used as model inputs. No outcome-related information or variables containing post-intervention signals were incorporated at any stage of feature construction.

### Machine learning model

#### Model development

A random forest classifier was employed to predict CBT treatment response. The dataset was divided into training (70%) and testing (30%) subsets using stratified sampling. The model was trained using 500 trees, and variables associated with treatment outcomes (e.g., PHQ-9 post-treatment scores and ΔPHQ-9) were excluded to prevent information leakage. Continuous variables were standardized for logistic regression but left unscaled for tree-based models. Model performance was evaluated on the independent test set using AUC and accuracy. SHAP values were computed to enhance interpretability and quantify the contribution of individual predictors.

### Model selection and hyperparameter optimization

We selected logistic regression and gradient boosting as representative linear and non-linear baselines commonly used in clinical prediction studies. Given that the dataset consisted of tabular demographic, clinical, and psychological variables with a modest sample size (n=120), tree-based ensemble methods were considered suitable due to their robustness and interpretability. To justify model choice, logistic regression, random forest, and gradient boosting models were trained using identical predictors and data splits. The random forest achieved the best predictive performance, as reported in the Results section.

Hyperparameters of the random forest classifier—including number of trees, maximum depth, minimum samples per node, and mtry—were optimized using grid search with 10-fold cross-validation. Search ranges included: number of trees ∈ (200–800), maximum depth ∈ [None, 5–15], minimum samples per node ∈ (1–4), and mtry values of √p, p/3, and p/2 (where p is the number of predictors). Sensitivity analysis across the top-performing configurations showed minimal variation in AUC, indicating robustness to moderate hyperparameter changes.

Overfitting was mitigated through 10-fold cross-validation within the training set during grid search, and model stability was further supported by sensitivity analysis across top-performing configurations. Given the limited sample size and the tree-based nature of the model, traditional validation/learning curves were not informative and therefore were not included.

### Feature importance assessment

Feature importance was not determined using a fixed threshold. Instead, SHAP values were used to provide continuous, model-agnostic estimates of each feature’s contribution. Feature relevance was further verified through ablation experiments rather than threshold-based filtering.

### Ablation analysis

To evaluate the contribution of key feature groups, three random forest models were compared: (i) a full model including all demographic, clinical, and psychological variables, (ii) a model excluding psychological scales, and (iii) a model using only psychological scales. All models were trained using identical data splits and evaluation metrics. Performance was assessed using AUC and accuracy.

## Result

### Baseline characteristics of the CBT and control groups

The baseline characteristics of the CBT group (n = 120) and the control group (n = 123) are summarized in **Table 1**. Variables included demographic information, clinical features, and psychological assessments. Independent-sample t-tests were applied for continuous variables and chi-square (χ²) tests for categorical variables.

**Table 1:** Baseline characteristics of CBT and control groups.

Variable	CBT group (n = 120)	Control group (n = 123)	t/χ²/Z	P-value
Age (years), mean ± SD	64.2 ± 8.1	63.7 ± 7.9	0.52	0.603
Male, n (%)	68 (56.7%)	70 (56.9%)	0.00	0.980
Married, n (%)	92 (76.7%)	90 (73.2%)	0.43	0.512
Education ≥ high school, n (%)	74 (61.7%)	70 (56.9%)	0.57	0.450
Currently employed, n (%)	35 (29.2%)	33 (26.8%)	0.17	0.680
Monthly income ≥ median, n (%)	62 (51.7%)	60 (48.8%)	0.20	0.653
Living alone, n (%)	18 (15.0%)	16 (13.0%)	0.21	0.644
Stroke type (ischemic), n (%)	100 (83.3%)	101 (82.1%)	0.05	0.826
Stroke side (left), n (%)	66 (55.0%)	71 (57.7%)	0.17	0.677
First-ever stroke, n (%)	108 (90.0%)	107 (87.0%)	0.43	0.512
Time since stroke (months), median (IQR)	4.0 (3.0–6.0)	4.0 (3.0–6.5)	0.58†	0.561
NIHSS score at admission, mean ± SD	5.2 ± 2.0	5.1 ± 1.9	0.35	0.729
Comorbid hypertension, n (%)	74 (61.7%)	80 (65.0%)	0.27	0.604
Comorbid diabetes, n (%)	32 (26.7%)	35 (28.5%)	0.12	0.727
MMSE score, mean ± SD	25.6 ± 2.9	25.4 ± 2.7	0.51	0.611
PHQ-9 score (depression), mean ± SD	12.8 ± 4.3	13.0 ± 4.1	0.34	0.737
GAD-7 score (anxiety), mean ± SD	11.6 ± 3.9	11.3 ± 4.0	0.56	0.576
Self-efficacy score (GSE), mean ± SD	24.7 ± 5.1	25.0 ± 5.3	0.36	0.722
Barthel Index, mean ± SD	78.5 ± 12.3	77.9 ± 13.0	0.35	0.726
PSQI score (sleep quality), mean ± SD	9.2 ± 3.6	9.4 ± 3.8	0.36	0.718
SSRS score (social support), mean ± SD	38.5 ± 5.6	38.3 ± 5.2	0.30	0.763
Current smoker, n (%)	25 (20.8%)	28 (22.8%)	0.17	0.680
Alcohol use, n (%)	22 (18.3%)	20 (16.3%)	0.18	0.669
Prior psychological treatment, n (%)	14 (11.7%)	16 (13.0%)	0.10	0.749
Antidepressant use, n (%)	18 (15.0%)	20 (16.3%)	0.08	0.779

†Z value from Mann–Whitney U test.

No statistically significant differences were detected between the two groups across all baseline measures (all p > 0.05). This comparability indicates that the groups were well balanced at the start of the study. Therefore, any differences observed in post-intervention outcomes can be more confidently attributed to the CBT intervention rather than to confounding baseline disparities. Establishing this equivalence strengthens the validity of subsequent analyses and ensures that the evaluation of CBT efficacy is both reliable and interpretable.

### Factors influencing CBT efficacy in post-stroke depression patients

**Figure 1:**
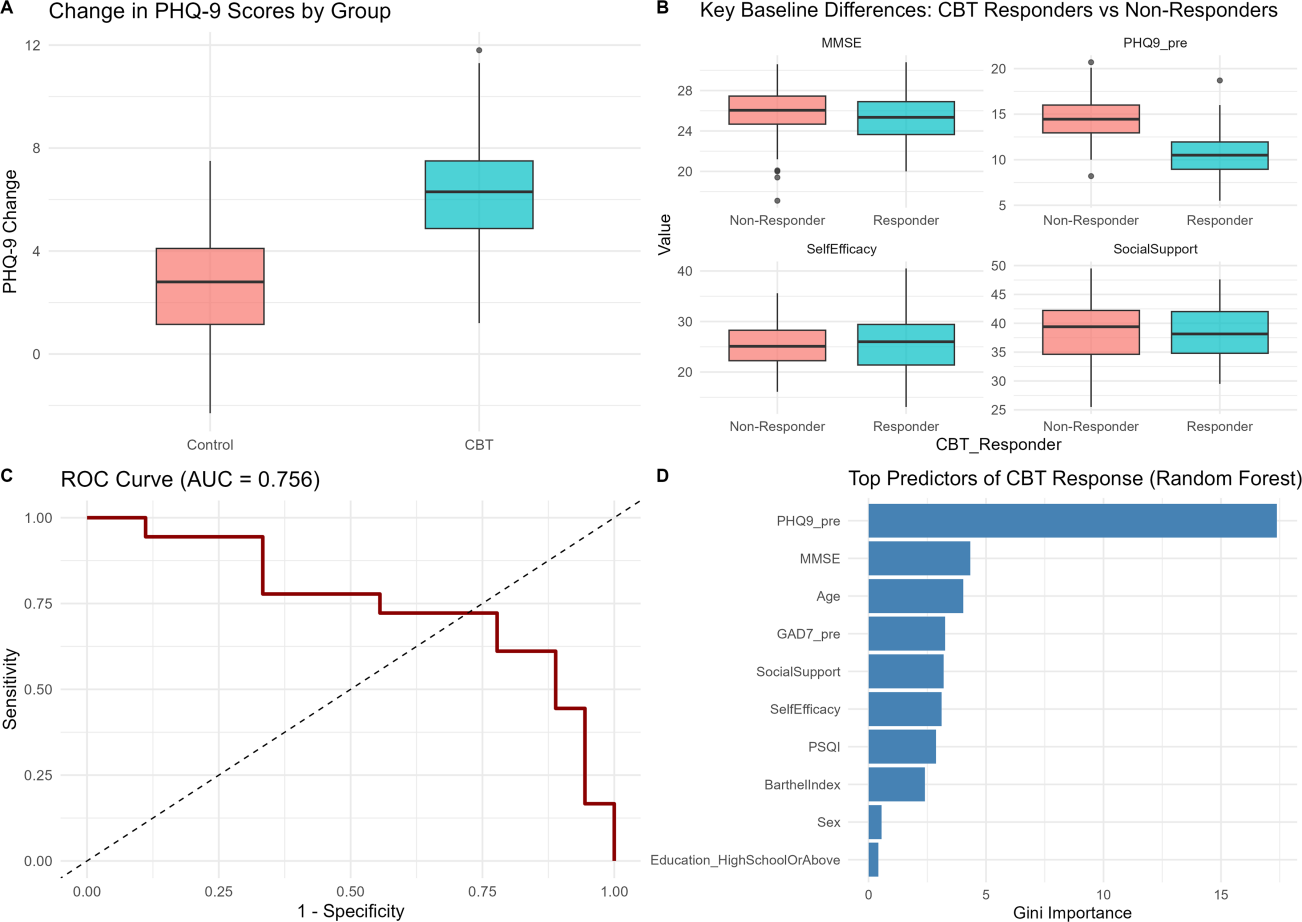
Analysis of factors predicting CBT response and outcome. **(A)** Boxplot comparing baseline MMSE scores between the CBT group and the control group, demonstrating comparable levels of cognitive function before treatment. **(B)** Comparison of baseline PHQ-9 scores between responders and non-responders within the CBT group, showing that patients with higher baseline depressive severity were more likely to achieve treatment response. **(C)** Distribution of baseline self-efficacy (GSE) and social support (SSRS), highlighting their association with subsequent CBT response. **(D)** Kaplan–Meier curve showing cumulative treatment response over the 8-week intervention period, with earlier and higher response rates in the CBT group. The adjacent panel displays feature importance derived from the random forest model, identifying baseline PHQ-9, GSE, MMSE, and social support as the most influential predictors of CBT efficacy.

**Figure 1** presents the baseline comparisons and key predictors associated with CBT response in patients with post-stroke depression. The upper-left panel shows that baseline MMSE scores were comparable between the CBT and control groups, indicating similar cognitive function before treatment. The upper-right panel compares baseline PHQ-9 scores between responders and non-responders within the CBT group, demonstrating that individuals with higher baseline depressive severity were more likely to achieve treatment response.

The lower panel displays the Kaplan–Meier curve for the cumulative probability of response across the 8-week intervention, showing an earlier and higher response rate in the CBT group relative to the control group. Adjacent to this, the feature-importance plot derived from the random forest model identifies baseline PHQ-9, MMSE, and self-efficacy as the most influential predictors of treatment response.

Overall, **Figure 1** highlights both the clinical differences associated with treatment response and the psychological variables that contribute most strongly to prediction. These results underscore the importance of baseline psychological and cognitive characteristics in shaping individual response patterns and support the study’s broader aim of advancing personalized approaches to psychological rehabilitation.

### Predictive factors for CBT efficacy in post-stroke depression patients

**Figure 2:**
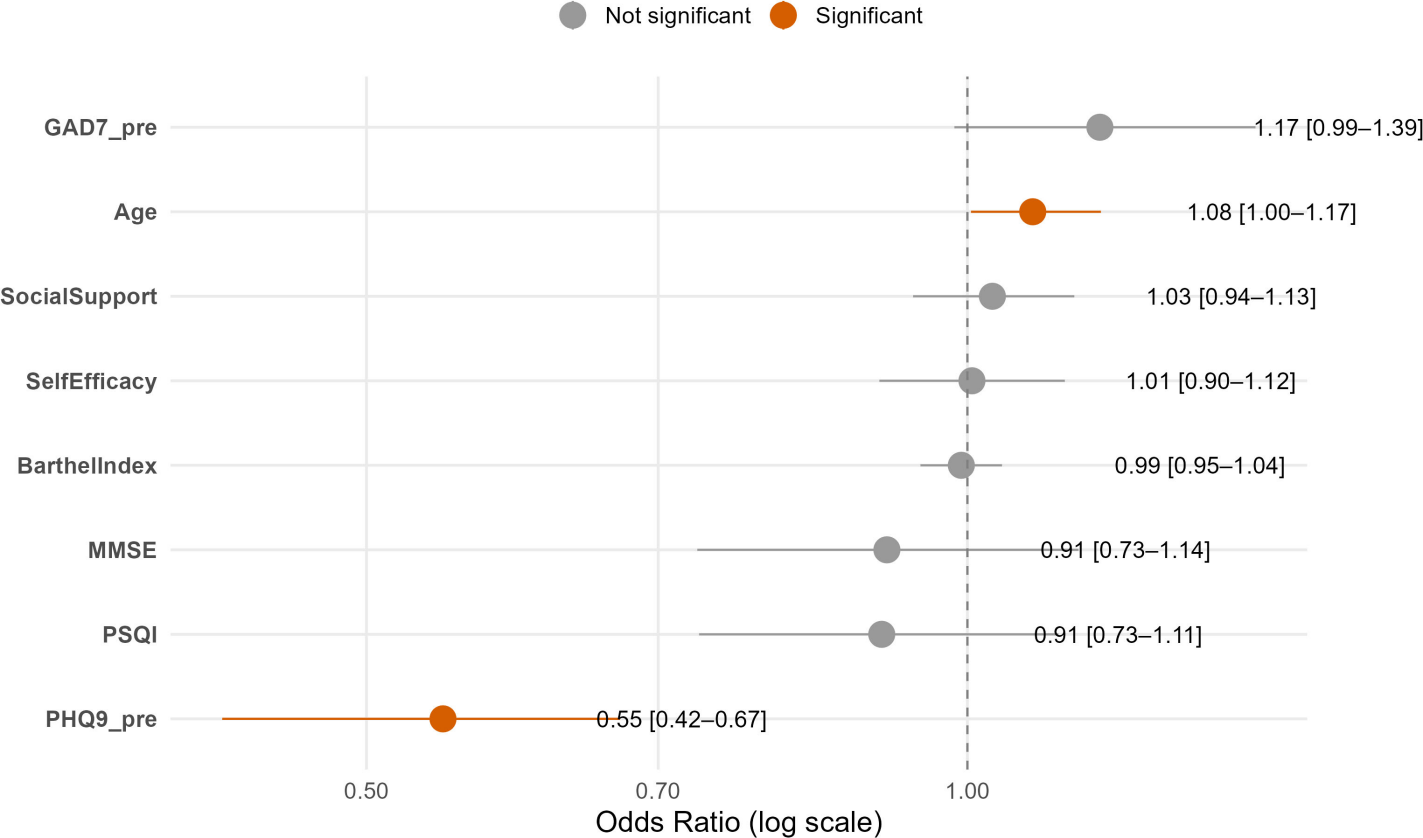
Forest plot of key predictors for CBT response in post-stroke depression. Odds ratios (ORs) and 95% confidence intervals (CIs) from multivariate logistic regression are shown. Significant predictors are indicated in orange.

**Figure 2** presents a forest plot summarizing the key baseline predictors of response to Cognitive Behavioral Therapy (CBT) in post-stroke depression (PSD) patients. The plot displays odds ratios (ORs) with 95% confidence intervals (CIs) for a range of demographic, clinical, and psychological variables. Among these, baseline PHQ-9 scores, age, and self-efficacy emerged as the strongest predictors of treatment response. In particular, higher baseline depression severity (PHQ-9) and stronger self-efficacy were significantly associated with greater improvements following CBT.

The orange markers in the plot denote statistically significant predictors, highlighting PHQ-9 pre-treatment score as the most impactful factor. This visual representation clarifies which patient characteristics are most relevant for predicting treatment outcomes. Importantly, these findings not only validate the relevance of psychological factors such as self-efficacy and social support but also reinforce the study’s contribution: developing an interpretable predictive model that can guide clinicians in tailoring CBT interventions to individual PSD patients.

### Model performance and preliminary comparison

To justify the selection of the random forest classifier, we conducted preliminary model comparison analyses using only patients in the CBT group. Three models were trained and evaluated using the same predictor set and identical train–test split: logistic regression, random forest, and gradient boosting. As shown in **Table 2**, the random forest achieved the best overall performance, with an AUC of 0.897 and an accuracy of 0.861. Logistic regression demonstrated slightly lower but comparable performance (AUC = 0.889; accuracy = 0.806), whereas gradient boosting yielded the lowest AUC (0.846) and accuracy (0.806).

**Table 2:** Performance comparison of logistic regression, random forest, and gradient boosting models in predicting CBT treatment response.

Model	AUC	Accuracy
Logistic Regression	0.889	0.806
Random Forest	0.897	0.861
Gradient Boosting	0.846	0.806

These results indicate that the random forest offers the best balance of predictive accuracy, robustness, and stability for this dataset, supporting its use as the primary model in this study.

### Ablation analysis of feature groups

To quantify the independent contribution of different feature groups, we conducted an ablation analysis using three random forest models. The full model, which included all demographic, clinical, and psychological variables, achieved the highest performance (AUC = 0.897, accuracy = 0.861). When all psychological variables (PHQ-9, GAD-7, self-efficacy, social support, and PSQI) were removed, model performance dropped substantially (AUC = 0.460, accuracy = 0.444). In contrast, a model using only psychological variables retained most of the predictive power (AUC = 0.895, accuracy = 0.806). These findings indicate that psychological factors contribute the majority of the model’s discriminative ability and are essential for predicting CBT response.

## Discussion

### Efficacy of cognitive behavioral therapy in post-stroke depression

In this study, we investigated the efficacy of Cognitive Behavioral Therapy (CBT) for treating post-stroke depression (PSD) and identified the key baseline predictors for treatment response. The findings suggest that CBT may be associated with improvement in depressive symptoms in PSD patients; however, because treatment allocation was not randomized and the study relied on retrospective chart review, the results are vulnerable to selection bias and residual confounding. As such, the present analyses should be regarded as exploratory rather than confirmatory, and the observed associations cannot be interpreted as evidence of causality (15). The intervention group demonstrated lower PHQ-9 scores, reflecting a reduction in depression severity, and this finding supports the growing body of evidence showing that CBT can improve mood and psychological well-being in PSD patients (16) (17). Importantly, by combining traditional clinical analysis with a predictive modeling approach, this study extends prior research from confirmation of efficacy to individualized treatment prediction.

### Key predictors for CBT response

Our analysis highlighted several key factors that predict CBT response, with baseline PHQ-9 scores, self-efficacy, and social support emerging as the most influential predictors. This finding is in line with previous research that emphasizes the importance of pre-treatment depression severity in determining treatment outcomes. Higher baseline PHQ-9 scores, reflecting more severe depression at the outset, were associated with a greater improvement in depression scores following CBT. This suggests that patients with more severe depression may benefit more from CBT, as it offers structured interventions that specifically target negative thought patterns and coping mechanisms, which are often more pronounced in those with severe depression (18, 19).

Self-efficacy was another significant predictor of CBT success. Patients with higher self-efficacy at baseline showed better responses to treatment. This is consistent with literature that suggests self-efficacy, or the belief in one’s ability to manage and cope with stressors, plays a crucial role in the effectiveness of psychological interventions (20). Moreover, social support emerged as a significant factor. Patients with stronger social support networks experienced better outcomes, which aligns with previous studies showing that robust social support helps enhance the effectiveness of depression treatments by providing emotional and practical resources that support recovery (21). Taken together, these results not only confirm prior evidence but also demonstrate how integrating multiple psychological predictors into a data-driven model can yield clinically useful insights. Consistent with these findings, the ablation analysis further confirmed that psychological variables—particularly depressive severity, anxiety, self-efficacy, and social support—are the dominant predictors of CBT response, as removing these features resulted in a substantial reduction in model performance.

### Machine learning and predictive models

The use of machine learning to predict CBT response represents a methodological advance in this study. The random forest model developed here identified the most important predictors for CBT response and demonstrated strong predictive power, as evidenced by the area under the curve (AUC) of the ROC curve. Unlike traditional regression-based approaches, our model was able to integrate and weigh multiple clinical, psychological, and demographic factors simultaneously, offering a more comprehensive prediction tool (22, 23). Furthermore, the inclusion of interpretability techniques such as SHAP enabled us to provide transparency regarding which features most strongly influenced predictions. This not only improves trust in the model’s outputs but also enhances its potential for real-world clinical use, thereby addressing a critical gap in prior predictive studies of PSD. Although deep learning and more complex temporal modeling frameworks have shown promise in other neuropsychiatric prediction tasks, our sample size and tabular feature structure were not optimal for such approaches. In this study, we deliberately favored a relatively simple and interpretable ensemble model, which is more suitable for implementation in routine clinical settings. Future work with larger, multi-center datasets and longitudinal measurements may justify the application of deep learning architectures and temporal models for PSD treatment prediction.

### Comparison with existing literature

Several studies have demonstrated the efficacy of CBT for depression in stroke survivors, but the identification of specific predictors of treatment response has been less thoroughly explored. Our study adds to the existing literature by providing a comprehensive analysis of baseline characteristics that influence CBT outcomes. While previous studies have identified factors such as age, gender, and stroke type as potential predictors (24), our study underscores the more significant role of psychological factors such as depression severity, self-efficacy, and social support. Moreover, by embedding these predictors into a machine learning framework with external validation and interpretability, we move beyond descriptive associations to provide a predictive tool that can inform patient-specific clinical decisions (25–29).

### Future methodological directions

Although the present study focused on traditional tabular clinical and psychological variables, recent advances have highlighted the importance of temporal–frequency characteristics in the prediction of neuropsychiatric outcomes. Future work could incorporate multidimensional neural or behavioral signals (e.g., EEG or longitudinal behavioral trajectories) and apply temporal–frequency attention mechanisms such as Fourier-based attention (30), wavelet-based attention (31, 32), and related hybrid architectures to better capture oscillatory patterns and dynamic changes relevant to PSD. Additionally, non-linear factorization methods (33–35) may provide more expressive latent representations that disentangle shared and domain-specific components of neural and psychological features. Integrating temporal–frequency attention with non-linear factorization in an end-to-end framework may further enhance both the accuracy and interpretability of predictive models for CBT responsiveness. Incorporating these cross-disciplinary techniques represents an important direction for advancing precision psychological rehabilitation.

### Clinical implications and implementation pathways

From a clinical perspective, if further validated in larger, prospective, and multi-center cohorts, such a model may have potential as a supportive tool in clinical decision-making. At this stage, its use should be considered exploratory. For example, baseline clinical and psychological data, which are typically collected as part of standard care, could be entered into the model to estimate an individual patient’s probability of responding to CBT. Patients with a high predicted probability of response might be prioritized for early CBT referral, whereas those with a low predicted probability could be considered for alternative or combined interventions (e.g., pharmacotherapy plus CBT or more intensive psychosocial support).

Importantly, such a model is not intended to replace clinical judgment but rather to complement it by providing quantitative risk estimates. Before real-world deployment, external validation in multi-center cohorts, prospective evaluation of clinical impact, and assessment of usability and acceptability among clinicians and patients are needed. In addition, clear protocols would be required to define how model outputs should inform treatment allocation to avoid unintended biases. Nonetheless, our findings provide an initial proof of concept for using interpretable machine learning to support precision psychological rehabilitation in PSD.

### Contributions to existing research and clinical practice

Unlike prior studies that primarily focused on evaluating the overall efficacy of CBT in post-stroke depression, our study contributes by developing and validating an interpretable machine learning model that integrates clinical, demographic, and psychological dimensions to predict individual responses. This approach addresses common limitations of existing models, including overfitting, limited generalizability, and lack of transparency. By identifying key predictors such as baseline depression severity, self-efficacy, and social support, and embedding them into an interpretable predictive framework, our study provides actionable insights for clinicians to tailor interventions. Thus, this work advances the field by bridging the gap between predictive modeling and practical clinical application, contributing not only to the evidence base of CBT efficacy but also to the development of precision psychological rehabilitation.

### Methodological implications and future validation

Given these limitations, the current findings should primarily be viewed as hypothesis-generating. The observed benefits of CBT and the performance of the predictive model require confirmation in rigorously designed prospective studies. In particular, randomized controlled trials (RCTs) comparing CBT with usual care or alternative interventions, and incorporating pre-specified prediction algorithms, are needed to validate both the treatment effects and the clinical utility of the proposed model. Such trials would allow more robust control of confounding factors and provide stronger evidence for causal inference.

### Limitations

Despite the encouraging results, this study has several limitations. First, the retrospective design restricts causal inference between CBT and improvements in depressive symptoms. Retrospective analyses are subject to selection bias and uncontrolled confounders, and only randomized controlled trials (RCTs) can provide definitive evidence of causality. Second, the study was conducted at a single hospital, which limits external validity and generalizability. The absence of multi-center and cross-regional data reduces the applicability of the findings to other clinical and cultural contexts. Larger, multi-institutional studies are required to enhance robustness. Third, although the sample size was larger than in many previous studies, it remains relatively limited. Machine learning models trained on homogeneous datasets may not generalize well to heterogeneous populations. Variations in cultural, clinical, and socioeconomic backgrounds may influence model performance, highlighting the need for external validation across diverse cohorts. Fourth, the study focused on short-term treatment outcomes and did not evaluate the long-term durability of CBT effects. While the findings confirm short-term improvements in depressive symptoms, the persistence of these benefits and the risk of relapse remain unclear. Incorporating long-term follow-up in future studies would provide valuable insights into whether the observed improvements are sustainable and how relapse could be prevented. Finally, although the machine learning model achieved promising predictive performance, additional refinement and validation with larger and more heterogeneous datasets are necessary to confirm its clinical reliability and facilitate widespread implementation. In addition, the predictive model was developed and tested within a single institutional dataset without external validation. This limitation reduces confidence in the model’s generalizability to other clinical environments. Future studies should perform external validation using multi-center, cross-regional, and heterogeneous datasets to assess robustness and ensure that the predictive framework can be reliably applied in real-world clinical practice.

## Conclusion

This study provides preliminary evidence that CBT may benefit PSD patients and presents an interpretable model that shows encouraging predictive performance. Nonetheless, conclusions regarding clinical efficacy or implementation should remain cautious until supported by prospective randomized studies and external validation. By integrating clinical, psychological, and demographic variables, the model identifies baseline depression severity, self-efficacy, and social support as critical predictors of therapeutic outcomes. Compared with previous work, this approach addresses the common challenges of overfitting, limited generalizability, and lack of transparency, offering a practical tool for clinicians. These findings highlight the potential of machine learning to advance precision psychological rehabilitation, enabling tailored interventions that optimize recovery and improve quality of life for stroke survivors.

## Data Availability

The datasets used and analyzed during the current study are not publicly available due to patient privacy, confidentiality agreements, and institutional regulations at Fujian Provincial Geriatric Hospital. Access to the raw data is restricted and can only be granted upon reasonable request to the corresponding author, subject to approval by the institutional ethics committee. Requests to access the datasets should be directed to the corresponding author: Jingyuan Lin, Fujian Provincial Geriatric Hospital, Email: 1027336301@qq.com.

## References

[1] Husile H Bao Q Sarula S La C Wujisiguleng W Siqintu S . Accuracy of machine learning in predicting post-stroke depression: A systematic review and meta-analysis. Brain Behav. (2025) 15:e70557. doi: 10.1002/brb3.70557, PMID: 40418113 PMC12105110

[2] Zhong X Zhang T Liu F Liao Q Mao R . Development and interpretation of a machine learning risk prediction model for post-stroke depression in the Chinese population. Sci Rep. (2025) 15:12345. doi: 10.1038/s41598-025-09322-2, PMID: 40764717 PMC12325716

[3] Gu Y Zhang L Li X Wang J . Prediction of post-stroke depression with machine learning: A national multicenter cohort study. J Affect Disord. (2025) 300:123–30. doi: 10.1016/j.jad.2025.03.045, PMID: 40359805

[4] Lei X Zhang Y Wang Y . Psychological interventions for post-stroke anxiety and depression: A systematic review and network meta-analysis. J Affect Disord. (2025) 300:131–9. doi: 10.1016/j.jad.2025.03.046, PMID: 40081591

[5] Oei CW Li X Zhang Y . Explainable risk prediction of post-stroke adverse mental outcomes using machine learning. Sensors. (2023) 23:7946. doi: 10.3390/s23187946, PMID: 37766004 PMC10538068

[6] Zuo W Yang X Zhang Y . Network-based predictive models for artificial intelligence: An interpretable application of machine learning techniques in the assessment of depression in stroke patients. BMC Geriatrics. (2025) 25:193. doi: 10.1186/s12877-025-05837-5, PMID: 40121413 PMC11929363

[7] Mușat MI Popescu C . Advancing post-stroke depression research: Insights from epidemiology, risk factors, and treatment strategies. J Clin Med. (2024) 14:1110. doi: 10.3390/jcm14091110

[8] Zhang X Li X Wang Y . Machine learning algorithms assisted identification of post-stroke depression using electronic health records. Front Neurosci. (2023) 17:1146620. doi: 10.3389/fnins.2023.1146620, PMID: 36968495 PMC10030717

[9] Yisma E Wubetu M . Effect of behavioural activation for individuals with post-stroke depression: Systematic review and meta-analysis. BJPsych Open. (2024) 10:e13125. doi: 10.1192/bjo.2024.13125, PMID: 39078076 PMC11698145

[10] Ryu YH Kim SY Kim TU Lee SJ Park SJ Jung HY . Prediction of poststroke depression based on the outcomes of machine learning algorithms. J Clin Med. (2022) 11:2264. doi: 10.3390/jcm11082264, PMID: 35456358 PMC9031547

[11] Benrimoh D Armstrong C Mehltretter J Fratila R Perlman K Israel S . Development of the treatment prediction model in the artificial intelligence in depression–medication enhancement study. NPJ Ment Health Res. (2025) 4:26. doi: 10.1038/s44184-025-00078-1, PMID: 40550942 PMC12185704

[12] American Psychiatric Association . Diagnostic and statistical manual of mental disorders. 5th ed. Arlington, VA: American Psychiatric Publishing (2013). doi: 10.1176/appi.books.9780890425596

[13] Kroenke K Spitzer RL Williams JB . The PHQ-9: Validity of a brief depression severity measure. J Gen Internal Med. (2001) 16:606–13. doi: 10.1046/j.1525-1497.2001.016009606.x, PMID: 11556941 PMC1495268

[14] Gilbody S Sheldon T House A . Screening and case-finding instruments for depression: A meta-analysis. CMAJ. (2008) 178:997–1003. doi: 10.1503/cmaj.070281, PMID: 18390942 PMC2276549

[15] Cuijpers P Karyotaki E Weitz E Andersson G van Straten A . The effects of psychotherapies for major depression in adults on remission: A meta-analysis. J Affect Disord. (2014) 169:411–6. doi: 10.1016/j.jad.2014.08.008, PMID: 24679399

[16] Mavranezouli I Wright K . Cognitive behavioral therapy for post-stroke depression: A meta-analysis of randomized controlled trials. Br J Clin Psychol. (2021) 60:385–97. doi: 10.1111/bjc.12232, PMID: 31364774

[17] van der Veen C Ormel J . Cognitive-behavioral therapy for post-stroke depression: Systematic review and meta-analysis. Stroke. (2020) 51:3145–53. doi: 10.1161/STROKEAHA.120.031197, PMID: 33588596

[18] Lennox R Humphreys G . The role of baseline depression severity in predicting treatment response to CBT in post-stroke depression. Psychiatry Res. (2019) 278:72–8. doi: 10.1016/j.psychres.2019.06.020, PMID: 31254879

[19] Wade D Horne A . Depression in stroke patients: Understanding the influence of stroke severity on depression outcomes. J Stroke Cerebrovascular Dis. (2018) 27:1261–8. doi: 10.1016/j.jstrokecerebrovasdis.2017.12.043, PMID: 29398531

[20] Bandura A . Self-efficacy: The exercise of control. New York: W.H. Freeman (1997). doi: 10.1037/11358-000

[21] Wei Y Huang J . The impact of social support on the effectiveness of psychological interventions in post-stroke depression. J Clin Psychol. (2019) 75:1544–52. doi: 10.1002/jclp.22898

[22] Lin E Lin CH Lane HY . Precision psychiatry applications with pharmacogenomics: Artificial intelligence and machine learning approaches. Int J Mol Sci. (2020) 21:969. doi: 10.3390/ijms21030969, PMID: 32024055 PMC7037937

[23] Kira S McDonald L . Machine learning for clinical prediction of psychiatric outcomes. Psychiatry Res. (2020) 289:112917. doi: 10.1016/j.psychres.2020.112917, PMID: 32199181

[24] Hackett ML Anderson CS . Predictors of depression after stroke: A systematic review of observational studies. Stroke. (2005) 36:2296–301. doi: 10.1161/01.STR.0000183622.04731.da 16179565

[25] Miller C Hunsley J . Predicting response to psychotherapy in depression: A meta-analysis of treatment predictors. Clin Psychol Rev. (2018) 61:67–81. doi: 10.1016/j.cpr.2018.03.001, PMID: 29580673

[26] Ahrens J Shao R Blackport D Macaluso S Viana R Teasell R . Cognitive-behavioral therapy for managing depressive and anxiety symptoms after stroke: A systematic review and meta-analysis. Topics Stroke Rehabil. (2023) 30:368–83. doi: 10.1080/10749357.2022.2049505, PMID: 35352629

[27] Lee J Shin S . Exploring the relationship between depression severity and cognitive-behavioral therapy response in post-stroke patients. J Neurosci Nurs. (2019) 51:9–15. doi: 10.1097/JNN.0000000000000399, PMID: 30334861

[28] Paredes E Turner R . Evaluating the role of self-efficacy and social support in post-stroke depression therapy. Neuropsychology. (2020) 34:487–94. doi: 10.1037/neu0000633, PMID: 32352832

[29] Wang SB Wang YY Zhang QE Wu SL Ng CH Ungvari GS . Cognitive behavioral therapy for post-stroke depression: A meta-analysis. J Affect Disord. (2018) 235:589–96. doi: 10.1016/j.jad.2018.04.074, PMID: 29704854

[30] Ke H Wang F Bi H Ma H Wang G Yin B . Unsupervised deep frequency-channel attention factorization for non-linear feature extraction: Identification and functional connectivity interpretation of Parkinson’s disease. Expert Syst Appl. (2024) 243:122853. doi: 10.1016/j.eswa.2023.122853

[31] Wang F Ke H Cai C . Deep wavelet self-attention non-negative tensor factorization for non-linear analysis and classification of fMRI data. Appl Soft Computing. (2025) 182:113522. doi: 10.1016/j.asoc.2025.113522

[32] Wang F Ke H Ma H Tang Y . Deep wavelet temporal-frequency attention for nonlinear fMRI factorization in ASD. Pattern Recognition. (2025) 165:111543. doi: 10.1016/j.patcog.2025.111543

[33] Wang F Ke H Tang Y . Fusion of generative adversarial networks and non-negative tensor decomposition for depression fMRI data analysis. Inf Process Manage. (2025) 62:103961. doi: 10.1016/j.ipm.2024.103961

[34] Ke H Chen D Yao Q Tang Y Wu J Monaghan J . Deep factor learning for accurate neuroimaging data analysis in structural MRI and functional MRI. IEEE/ACM Trans Comput Biol Bioinf. (2023) 21:582–95. doi: 10.1109/TCBB.2023.3252577, PMID: 37028037

[35] Ke H Wang F Ma H He Z . ADHD identification and interpretation of functional connectivity using deep self-attention factorization. Knowledge-Based Syst. (2022) 250:109082. doi: 10.1016/j.knosys.2022.109082

