# Correction: Toward precision psychological rehabilitation: predicting CBT efficacy in post-stroke depression using machine learning

**DOI:** 10.3389/fpsyt.2026.1787831

**Published:** 2026-01-28

**Authors:** Jingyuan Lin, Jiansong Yu

**Affiliations:** 1 Department of Rehabilitation Medicine, Fujian Provincial Geriatric Hospital, Fuzhou, China; 2 Department of Rehabilitation Medicine, Taizhou Hospital of Zhejiang Province Affiliated to Wenzhou Medical University, Taizhou, China

**Keywords:** cognitive behavioral therapy (CBT), machine learning, post-stroke depression (PSD), predictive modeling, retrospective analysis

Author Jiansong Yu was erroneously omitted as equal contributing author.

The original version of this article has been updated.

